# Glucocerebrosidase Gene Therapy Induces Alpha-Synuclein Clearance and Neuroprotection of Midbrain Dopaminergic Neurons in Mice and Macaques

**DOI:** 10.3390/ijms22094825

**Published:** 2021-05-01

**Authors:** Diego Sucunza, Alberto J. Rico, Elvira Roda, María Collantes, Gloria González-Aseguinolaza, Ana I. Rodríguez-Pérez, Iván Peñuelas, Alfonso Vázquez, José L. Labandeira-García, Vania Broccoli, José L. Lanciego

**Affiliations:** 1 Centro de Investigación Médica Aplicada (CIMA), Department of Neurosciences, Universidad de Navarra, 31008 Pamplona, Spain; dsucunza@alumni.unav.es (D.S.); eroda@unav.es (E.R.); 2 Centro de Investigación Biomédica en Red de Enfermedades Neurodegenerativas (CiberNed), 28031 Madrid, Spain; ggasegui@unav.es (G.G.-A.); anai.rodriguez@usc.es (A.I.R.-P.); joseluis.labandeira@usc.es (J.L.L.-G.); 3 Instituto de Investigación Sanitaria de Navarra (IdiSNA), 31008 Pamplona, Spain; mcollant@unav.es (M.C.); ipenuelas@unav.es (I.P.); alfonso.vazquez.miguez@cfnavarra.es (A.V.); 4 Department of Nuclear Medicine, Clínica Universidad de Navarra, 31008 Pamplona, Spain; 5 Centro de Investigación Médica Aplicada (CIMA), Department of Gene Therapy, Universidad de Navarra, 31008 Pamplona, Spain; 6 Research Center for Molecular Medicine and Chronic Diseases (CIMUS), Universidad de Santiago de Compostela, 15782 Santiago de Compostela, Spain; 7 Complejo Hospitalario de Navarra, Department of Neurosurgery, Servicio Navarro de Salud, 31008 Pamplona, Spain; 8 San Raffaele Scientific Institute, Stem Cell and Neurogenesis Unit, Division of Neuroscience, 20132 Milano, Italy; broccoli.vania@hsf.it

**Keywords:** GBA1, adeno-associated viral vectors, synucleinopathies, neuroprotection, Parkinson’s disease

## Abstract

Mutations in the GBA1 gene coding for glucocerebrosidase (GCase) are the main genetic risk factor for Parkinson’s disease (PD). Indeed, identifying reduced GCase activity as a common feature underlying the typical neuropathological signatures of PD—even when considering idiopathic forms of PD—has recently paved the way for designing novel strategies focused on enhancing GCase activity to reduce alpha-synuclein burden and preventing dopaminergic cell death. Here we have performed bilateral injections of a viral vector coding for the mutated form of alpha-synuclein (rAAV9-SynA53T) for disease modeling purposes, both in mice as well as in nonhuman primates (NHPs), further inducing a progressive neuronal death in the substantia nigra pars compacta (SNpc). Next, another vector coding for the GBA1 gene (rAAV9-GBA1) was unilaterally delivered in the SNpc of mice and NHPs one month after the initial insult, together with the contralateral delivery of an empty/null rAAV9 for control purposes. Obtained results showed that GCase enhancement reduced alpha-synuclein burden, leading to improved survival of dopaminergic neurons. Data reported here support using GCase gene therapy as a disease-modifying treatment for PD and related synucleinopathies, including idiopathic forms of these disorders.

## 1. Introduction

The GBA1 gene is located in chromosome 1 of the human genome and encodes the lysosomal enzyme glucocerebrosidase (GCase) that hydrolyzes glucosylceramide down to glucose and ceramide. In the past few years, accumulating evidence was provided sustaining a direct genetic link between GBA1 homo- and heterozygous mutations and increased incidence of synucleinopathies, including Parkinson’s disease (PD) and dementia with Lewy bodies (DLB) [1,2,3,4]. It has been estimated that 7–10% of PD patients harbor GBA1 mutations [2,5], these patients facing a slight earlier disease onset together with a more aggressive disease course, including a faster progression to dementia, a higher prevalence of neuropsychiatric symptoms, autonomic dysfunction and increased risk of mortality [6]. Moreover, it is worth noting that up to 1% of individuals in the general population are heterozygous GBA1 mutation carriers [7], this incidence increasing to 6% in the Ashkenazi Jewish population [8]. Furthermore, it has also been postulated a relationship between the type of GBA1 mutation variant and the risk of PD and dementia. In this regard, the N370S mutation variant (currently known as p.Asn409Ser) seems to be closer to PD, whereas the L444P mutation (renamed as p.Leu483Pro) is more likely associated with a faster progression to dementia [9].

Although conclusive experimental evidence disclosing the mechanisms through which GCase regulates alpha-synuclein (α-syn) homeostasis—and vice versa—is still lacking, it has been postulated the presence of a bidirectional loop [10]. Bearing in mind that most enzyme-related inherited disorders are due to a loss of function [11], a deficit in GCase enzymatic activity would promote α-syn aggregation, the neuropathological hallmark characterizing PD and DLB. Conversely, α-syn aggregation in itself (e.g., without GBA1 mutations) induces GCase loss-of-function, and indeed GCase deficiency has been found in the brain, cerebrospinal fluid and blood of patients suffering from idiopathic PD [3,12,13], such GCase deficiency correlating with an increased α-syn burden. Moreover, it is also worth noting the presence of a natural decline in GCase expression levels with aging [14].

Accordingly, approaches aimed at enhancing GCase activity will likely have therapeutic potential for the treatment of PD patients, with and without GBA1-associated mutations. In this regard, several different therapeutics choices are currently under development, these including enzymatic replacement, GCase chaperones and substrate reduction. Finally, another choice for GCase enhancement is represented by gene therapy approaches taking advantage of different types of adeno-associated viral vectors (rAAVs) encoding the GBA1 gene. In this regard, rAAV-mediated overexpression of GCase has managed to reduce α-syn burden, further inducing neuroprotection both in animal models of PD [15,16] and in murine models of Gaucher disease [17,18].

Here, we have implemented a gene therapy strategy to enhance GCase activity in mice and nonhuman primate (NHP) models of PD-like synucleinopathy by taking advantage of the intraparenchymal focused delivery into the substantia nigra pars compacta (SNpc) of rAAVs coding for the GBA1 gene. Compared to previous methods, our goal was to provide evidence supporting using viral-mediated GCase enhancement as a neuroprotective treatment for synucleinopathies once neurodegenerative changes followinfg progressive α-syn aggregation are already ongoing, but before reaching a non-returning point.

## 2. Results

Obtained data suggest that rAAV-mediated GCase enhancement can be considered as a feasible strategy for the treatment of initial stages of synucleinopathies. By taking advantage of two animal models (mice and NHPs) of rAAV-induced α-syn aggregation, the conducted approach revealed that using rAAV9-GBA1 resulted in a substantial clearance of α-syn aggregates, further leading to dopaminergic cell neuroprotection.

### 2.1. Experiments Conducted in Mice

Mice received a bilateral injection of a neurospecific rAAV9-SynA53T into the SNpc, one site in each hemisphere. Four weeks later, animals were injected into the right SNpc with a rAAV9 coding for the GBA1 gene under the control of the constitutive promoter GusB, together with an empty rAAV9 (rAAV9-null) into the left SNpc for control purposes. Animals were sacrificed four weeks later (e.g., eight weeks after initial deliveries of rAAV9-SynA53T).

**Figure 1:**
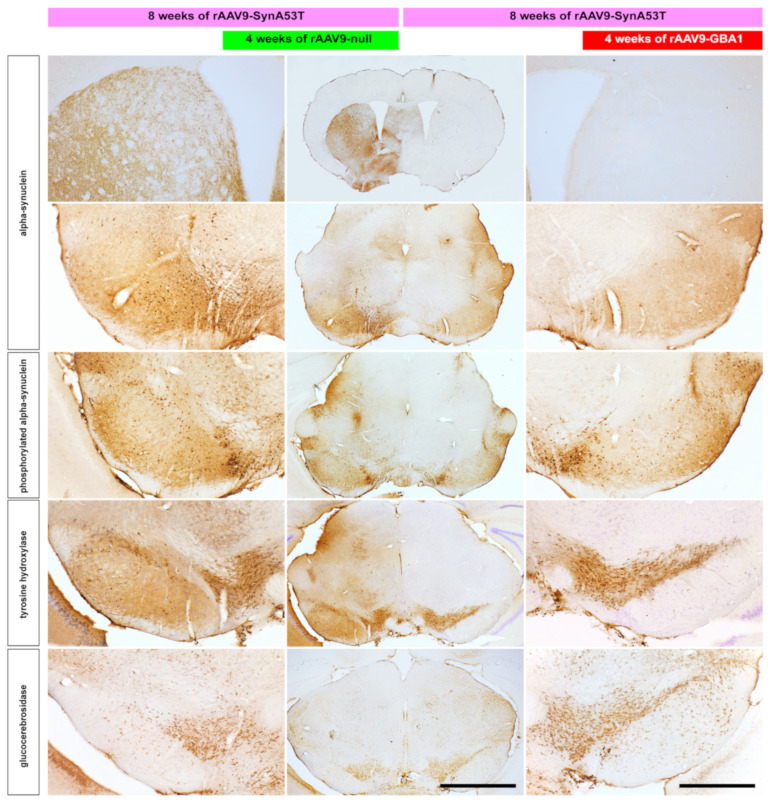
Immunohistochemical stains conducted in mice. Coronal sections through the mice brain taken at the level of caudal mesencephalon and striatum. rAAV-mediated enhancement of GCase activity in the right SNpc resulted in an almost complete clearance of α-syn burden, both at the level of the SNpc as well as in the striatum when compared to the contralateral SNpc where a control-null vector was delivered. Reduction in α-syn burden exerted a neuroprotective effect on the number of TH+ neurons in the SNpc, in keeping with enhanced expression of GCase. Scale bar is 1000 μm for insets and 2000 μm in low-magnification panels (4000 μm when considering the panel showing a coronal section through the striatum).

Conducted immunohistochemical (IHC) stains revealed strong immunoreactivity for α-syn both in the left SNpc (first, injected with rAAV9-SynA53T, later followed by rAAV9-null delivery) as well as in the ipsilateral striatum, and a similar staining pattern was also observed for phosphorylated α-syn. In parallel, a clear reduction was noticed concerning both tyrosine hydroxylase (TH) and glucocerebrosidase (GCase) IHC stains. By contrast, at the level of the right SNpc (e.g., the one initially injected with rAAV9-SynA53T and later on with rAAV9-GBA1), α-syn immunoreactivity was clearly reduced to the point that only very few SNpc neurons remained immunopositive for α-syn, whereby α-syn stain was almost completely absent throughout all striatal levels analyzed. Furthermore, rAAV-mediated enhancement of GCase substantially reduced phosphorylated α-syn in the right SNpc, where only dopaminergic neurons in the ventral tegmental area remained positive. Most importantly, the reduction of α-syn burden exerted a substantial neuroprotective effect on dopaminergic neurons in the right SNpc, as seen by TH stain. This is in keeping with the more abundant expression of GCase observed in the right vs. the left SNpc. Illustrative examples of the conducted IHC stains are provided in Figure 1.

**Figure 2:**
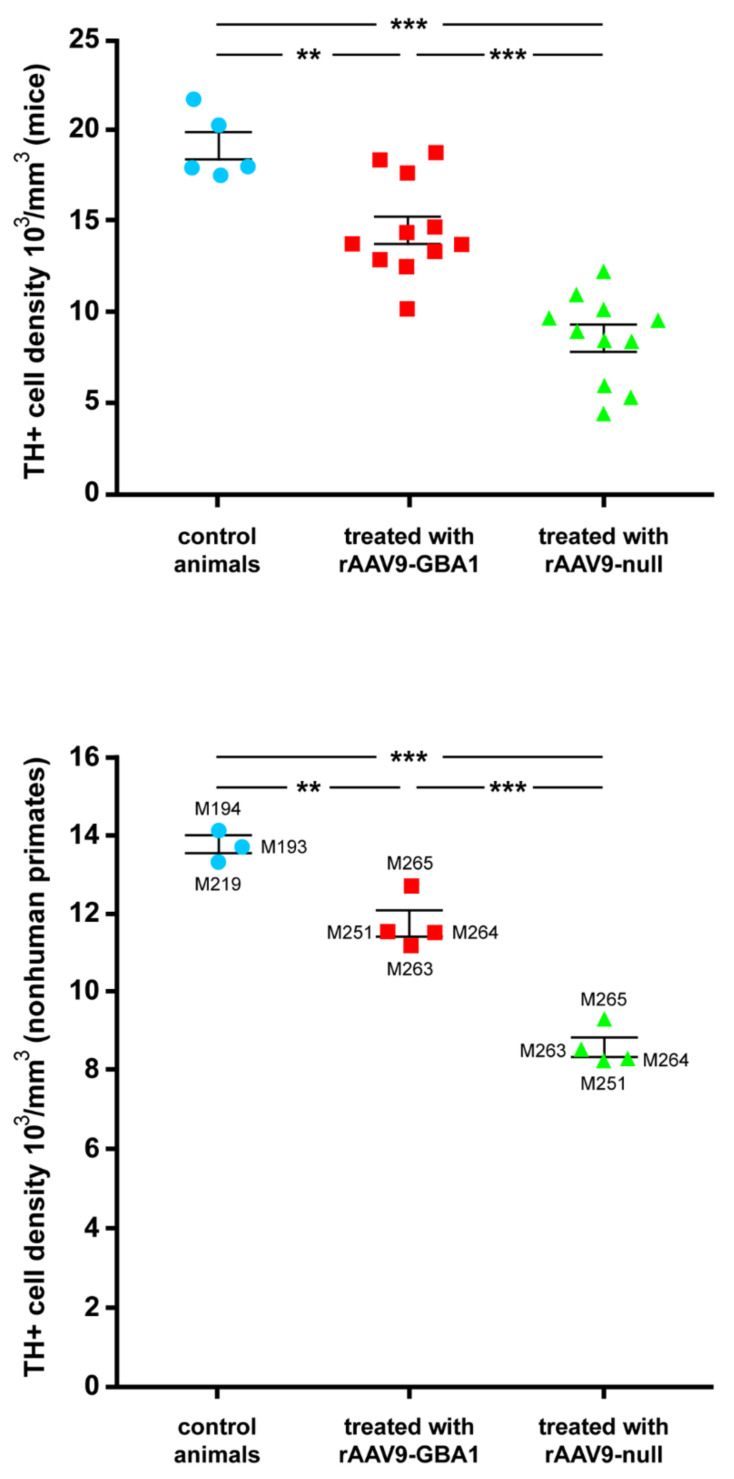
Unbiased stereological estimation of TH-ir neurons in the SNpc. Top: results gathered from mice experiments. The mean baseline density of TH-ir neurons was calculated in 5 naïve mice (blue dots and bar) and used for comparison purposes of mice treated with rAAV9-GBA1 (red dots and bar) and with rAAV9-null (green dots and bar). Eight weeks post-delivery of rAAV9-SynA53T, an average loss of TH+ cells of 55.1% was found in the SNpc upon treatment with the control vector, contrasting with 23.6% of TH+ neuronal density found in the right SNpc (e.g., the one treated with rAAV9-GBA1). Bottom: stereological estimation of the density of TH+ neurons in the nonhuman primate SNpc. Three control naïve NHPs were used to estimate the baseline TH+ neuronal density (blue dots and bar). Within the right SNpc (treated with rAAV9-null vector), a TH+ cell loss of 38.1% was found with a follow-up of 3 months post-injection of rAAV9-SynA53T, whereby cell loss was reduced to 14.9% on average in the SNpc injected with rAAV9-GBA1. Statistical comparisons were found to be highly significant for all groups (*p* < 0.05 for comparisons between control groups and animals treated with rAAV9-GBA1; *p* < 0.001 for comparisons between treated and untreated animal groups; *p* < 0.001 for comparisons between control groups and animals treated with rAAV9-null). **: *p* < 0.01; ***: *p* < 0.001.

Unbiased stereological estimation of the density of TH+ neurons in the left and right SNpc was conducted and compared to the TH+ mean neuronal density measured in a separate group of five naïve animals that were used for control purposes. Obtained data estimated a 55.1% of mean neuronal death in the left SNpc 8 weeks after the initial insult with rAAV9-SynA53T, contrasting with a 23.6% reduction in TH+ neuronal densities observed in the right SNpc, i.e., the one treated with rAAV9-GBA1 instead of rAAV9-null (Figure 2).

### 2.2. Experiments Conducted in NHPs

Data obtained in mice provided proof-of-principle and laid the groundwork to design a similar approach in NHPs. Accordingly, 4 NHPs were bilaterally injected with a neurospecific rAAV9-SynA53T into the SNpc. Two sites spaced 2 mm in between within the SNpc of each hemisphere. Four weeks later, animals were injected into the left SNpc with a rAAV9 coding for the GBA1 gene under the control of the constitutive promoter GusB (two deposits) and with an empty rAAV9 (rAAV-null) into the right SNpc for control purposes (two deposits). Animals were sacrificed 8 weeks later (e.g., 12 weeks after initial deliveries of rAAV9-SynA53T).

#### 2.2.1. MicroPET Neuroimage Studies

**Figure 3:**
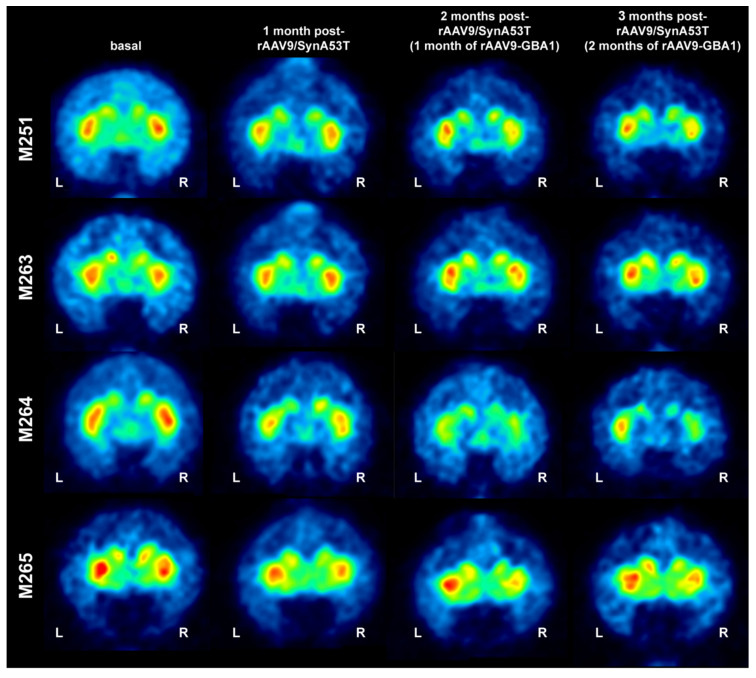
MicroPET follow-up of NHPs. Representative coronal images taken at the level of the post-commissural caudate and putamen nuclei for all the 4 NHPs included in the study. MicroPET scans with ^11^C-DTBZ were taken at baseline, and 1, 2 and 3 months post-bilateral delivery of rAAV9-SynA53T. Compared to baseline radiotracer uptake levels, a bilateral decline is observed in all animals one month post-injection of rAAV9-SynA53T. One month later (e.g., 2 months posttreatment with rAAV9-SynA53T and 1 month post-injection of rAAV9-GBA1 in the left SNpc and rAAV9-null in the right SNpc), an asymmetrical pattern of radiotracer uptake is noticed in all animals with the only exception of M263. MicroPET scans were repeated one month later (3 months of rAAV9-SynA53T, including 2 months of rAAV9-GBA1 and rAAV-null), evident left vs. right differences in radiotracer uptake were observed in all animals. When compared to baseline levels, observed differences with a follow-up of 3 months failed to reach statistical significance.

The density of dopaminergic innervation of the caudate and putamen nuclei was assessed in vivo by neuroimage MicroPET scanning using the radiotracer ^11^C-dihydrotetrabenazine (^11^C-DTBZ; a selective VMAT2 ligand). Scans were performed at baseline and 4, 8, and 12 weeks post-injection of rAAV9-SynA53T. All animals showed a bilateral reduction in the ^11^C-DTBZ-binding potential four weeks after delivery of rAAV9-SynA53T, although to a variable interindividual extent. Four weeks after delivery of rAAV9-GBA1 (left SNpc) and rAAV-null (right SNpc), 3 out of 4 animals showed an asymmetrical pattern of ^11^C-DTBZ uptake that was confirmed at eight weeks’ time point for all animals (e.g., 12 weeks post-bilateral injection of rAAV9-SynA53T). However, although there was a clear trend supporting left vs. right asymmetries in radiotracer uptake (Figure 3) when compared to baseline levels, observed differences failed to reach statistical significance.

#### 2.2.2. GCase Expression Levels

**Figure 4:**
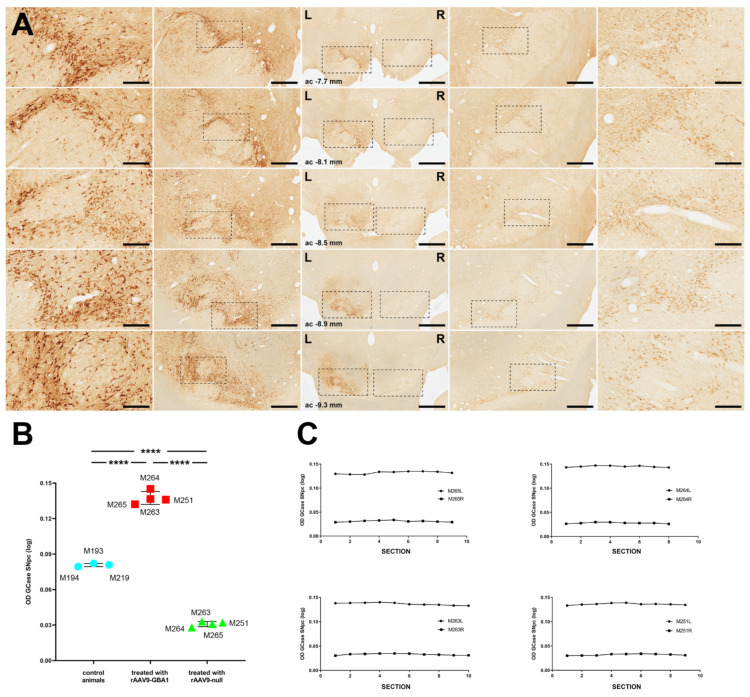
Immunohistochemical detection of GCase in the SNpc of NHPs. (**A**) Eight weeks after the delivery of two deposits of 10 μL of rAAV9-GBA1 into the left SNpc and 10 μL of AAV9-null into the right SNpc, high levels of GCase expression were found in the left SNpc (e.g., the one injected with the therapeutic vector). By contrast, only baseline GCase levels were detected in the right SNpc. Furthermore, it is worth noting that the entire extent of the left SNpc was successfully transduced with rAAV9-GBA1 since GCase+ neurons were found throughout the full rostrocaudal extent of the SNpc, ranging from rostral SNpc levels (ac—7.7 mm) to the caudalmost territories (ac—9.3 mm). Scale bars are 3000 μm for A-G (low-magnification panels), 1000 μm for panels A1-G1 and A3-G3, and 300 μm for high-magnification insets (panels A2-G2 and A4-G4). (**B**) Optical densities of GCase stains as measured in the side treated with rAAV9-GBA1 (left SNpc; red), in the side treated with the control vector rAAV9-null (right SNpc; green), compared to baseline GCase optical densities observed in three naïve animals at the level of the SNpc. (**C**) Illustrations showing that enhanced GCase expression levels in the left vs. right SNpc are maintained throughout the entire rostrocaudal extent of the SNpc. ****: *p* < 0.0001.

The conducted IHC stains of NHP brain samples revealed very similar results compared to those formerly obtained in mice. The intranigral delivery of rAAV9-GBA1 resulted in increased expression of GCase protein in the SNpc. Neurons showing enhanced GCase protein levels were noticed throughout the whole rostrocaudal extent of the SNpc (e.g., between 7.7 and 9.3 mm caudal to the anterior commissure; Figure 4A). Neurons located in the left SNpc (e.g., the one injected with rAAV9-GBA1) displayed stronger GCase immunoreactivity than the contralateral SNpc, i.e., the side injected with the rAAV9-null vector (Figure 4A). In other words, although the SNpc is known to be one of the brain areas characterized by showing high baseline levels of GCase expression [19], the obtained rAAV9-GBA1-mediated enhancement of GCase expression in the left SNpc was substantially above contralateral levels. As an indirect estimation of GCase expression levels, optical densities of GCase IHC were measured in the left vs. right SNpc and compared to baseline staining intensities gathered from 3 naïve animals. Obtained data revealed that the delivery of rAAV9-GBA1 into the left SNpc increased GCase levels up to 77.4% on average compared to the levels observed in the right SNpc and by 41.03% above baseline levels (Figure 4B). Furthermore, in keeping with the bidirectional loop sustaining the interplay between GCase and α-syn [10], rAAV-mediated expression of α-syn into the right SNpc (e.g., the one treated with the control-null vector) revealed a GCase optical density of 61.67% below baseline GCase expression levels observed in naïve animals (Figure 4B). Finally, it is also worth noting that the observed increases in GCase optical densities were maintained throughout the full rostrocaudal extent of the SNpc in all animals treated with rAAV9-GBA1 and with rAAV9-null (Figure 4C).

#### 2.2.3. rAAV-Mediated GCase Enhancement Is Linked to α-Syn Clearance

**Figure 5:**
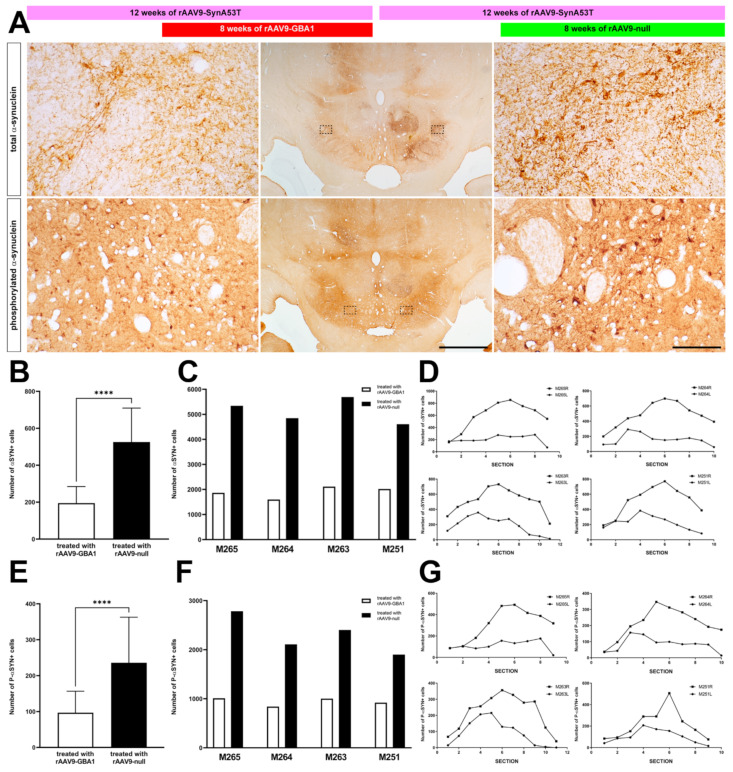
Immunohistochemical detection of α-syn in NHPs. (**A**) Coronal sections through the caudal mesencephalon taken at the level of the SNpc showing the obtained stains for total α-syn (top panels) and phosphorylated α-syn (bottom panels). Even at low magnification, there is a clear noticeable left vs. right difference in the obtained staining patterns. High-magnification insets showed a substantial reduction of α-syn burden in neurons and neurites within the left SNpc (e.g., the one treated with rAAV9-GBA1; left panels) when compared to the right SNpc (right panels). Scale bar is 2000 μm for low-magnification photomicrographs and 200 μm in insets. (**B**) Histogram showing reduced α-syn burden (e.g., number of α-syn+ cells) in the left SNpc compared with the right SNpc (white and black bars, respectively). (**C**) data gathered from each animal when considered individually. (**D**) The rostrocaudal extent of α-syn+ cells in the left vs. the right SNpc for all treated animals. (**E**) Histogram showing the reduction in phospho-α-syn+ cells in the left SNpc (e.g., the one treated with rAAV9-GBA1; white bar) compared to the SNpc treated with the control-null vector (black bar). (**F**) Data taken from each animal and (**G**) Rostrocaudal distribution of phospho-α-syn+ cells in all macaques. ****: *p* < 0.0001.

Twelve weeks post-bilateral delivery of rAAV9-SynA53T, expression of both total and phosphorylated α-syn was observed in the cell bodies and neurites of neurons located in the right SNpc. Expression levels of α-syn were high enough to be visible even under low-power magnification. By contrast, when considering the left SNpc, i.e., the one treated with rAAV-GBA1, the number of α-syn+ neurons is markedly reduced, and indeed only a moderate number of neurons still remained immunopositive for α-syn (Figure 5A). Obtained quantitative data revealed that α-syn burden in the left SNpc was reduced by 62.89% when compared to the right SNpc (Figure 5B,C). A similar trend also applies when considering the number of neurons expressing phosphorylated α-syn, where a reduction of up to 58.37% on average was observed in the left SNpc vs. the right SNpc (Figure 5E,F). Moreover, the positive effect of rAAV-mediated GCase enhancement on α-syn clearance was maintained throughout the whole rostrocaudal extent of the SNpc (Figure 5D,G).

#### 2.2.4. Neuroprotection of the Nigrostriatal System

**Figure 6:**
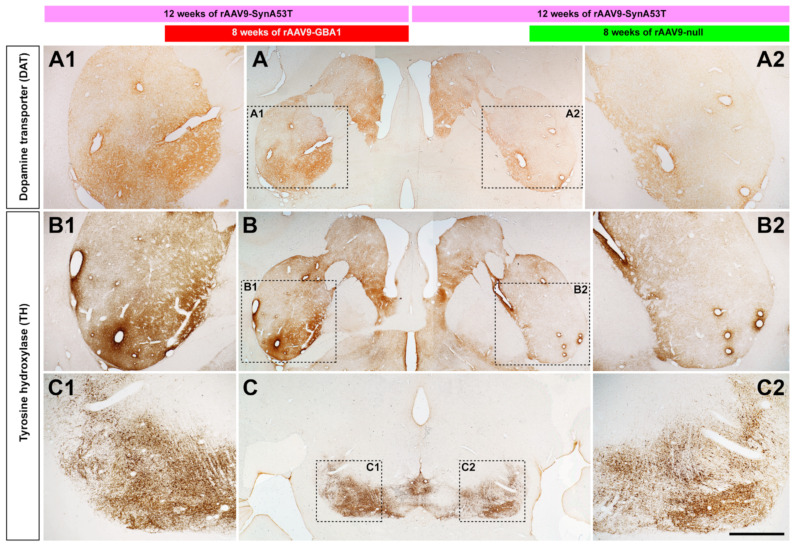
Immunohistochemical detection of dopaminergic markers in NHPs. Panels A and B are coronal sections taken at the level of the post-commissural caudate and putamen nuclei showing the staining pattern for the dopaminergic transporter (DAT) and TH. Panels **A**, **A1** and **A2** show a higher staining intensity obtained for DAT in the left striatum (e.g., the one innervated by the left SNpc where rAAV9-GBA1 was delivered) when compared to the right striatum (e.g., the one located ipsilateral to the right SNpc, injected with the control vector). Panels **B**, **B1** and **B2** were taken from an adjacent section and showed that the TH staining patterns were very similar to the ones of DAT. Panels **C**, **C1** and **C2** were taken at the level of the SNpc and illustrate the left vs. right differences in TH-immunoreactive profiles. Even at low magnification, it can easily be observed that the treatment with rAAV9-GBA1 (left SNpc, **C1** inset) resulted in better preservation of the number of TH+ neurons when compared to the right SNpc (the latter treated with the control vector; **C2** inset). Scale bar is 4000 μm for low-magnification images and 2000 μm for insets.

The obtained reduction in α-syn burden, linked to GCase overexpression, induced a neuroprotective effect in the dopaminergic system. Both the number of TH+ neurons in the left SNpc and the density of TH+ terminals in the ipsilateral caudate and putamen nuclei are clearly higher when compared to the right SNpc, i.e., the one treated with the empty-null vector (Figure 6). To what extent the dopaminergic system was better preserved in the side treated with rAAV9-GBA1 is best exemplified by the staining pattern observed for the dopamine transporter DAT (Figure 6).

Unbiased stereological estimation of the density of TH+ neurons in the left and right SNpc was conducted and compared to the TH+ mean neuronal density measured in a separate group of 3 naïve NHPs that were used for control purposes. Obtained data estimated a 39.1% of mean TH+ cell loss in the right SNpc 12 weeks after the initial insult with rAAV9-SynA53T, contrasting with a 14.9% on average reduction in TH+ neuronal densities observed in the left SNpc, i.e., the one treated with rAAV9-GBA1 instead of rAAV9-null (Figure 2).

## 3. Discussion

Evidence was provided here further supporting using GCase gene therapy as a disease-modifying strategy in the early stages of synucleinopathies in two different animal models.

### 3.1. Considerations on rAAV-Mediated Animal Models of PD-Like Synucleinopathies

Although neurotoxin-based mammalian animal models of PD have settled the basis for most of our current understanding of basal ganglia function and dysfunction [20,21], these models failed to recapitulate the main neuropathological hallmarks that typically characterize PD (e.g., dopaminergic cell death driven by α-syn aggregation).

The use of rAAVs coding for α-syn for the purpose of PD modeling in mammalian species is currently in the spotlight, and indeed, rAAVs are versatile enough to collectively represent an appropriate choice for disease modeling. When designed this way, the intraparenchymal delivery of different rAAV serotypes carrying the α-syn gene both in rodents and in NHPs showed a variable degree of dopaminergic damage upon the time-dependent aggregation of α-syn [22]. In this regard, using rAAVs coding for either wild-type or mutated forms of α-syn in rats resulted in a time-dependent loss of up to 80% of dopaminergic neurons with a follow-up of 8 weeks [23], this approach is later validated by independent groups [24,25] showing a 52% of dopaminergic cell loss with a shorter follow-up period of 3 weeks. A similar approach conducted in marmosets based on using rAAV2 showed a variable 30–60% loss of dopaminergic neurons together with well-established α-syn neuropathology [26]. Furthermore, when using rAAV5 instead of rAAV2 for triggering α-syn aggregation in marmosets, a reproducible cell loss above 40% was observed in the injected marmosets [27]. Considering Old World monkeys (*M. fascicularis*), using chimeric rAAV1/2 coding for mutated human α-syn resulted in a 50% reduction of TH+ neurons in the SNpc 17 weeks post-rAAV delivery [28]. Although work presented here was not designed for these purposes, obtained findings also support using the intraparenchymal delivery of rAAVs encoding SynA53T for disease modeling purposes in cynomolgus macaques, bearing in mind that a dopaminergic cell loss of up to 39% was observed with a follow-up period of 12 weeks in the SNpc first, injected with rAAV9-SynA53T and later on with rAAV9-null. The use of different technologies, including the choice of rAAV serotypes and promoters, viral titration, number of injections and follow-up period, may account for the observed differences in terms of neuronal death.

It is also worth noting that the rAAV field is a quickly changing scenario, with new arrivals being incorporated at a breath-taking speed [29]. Different viral vector capsids, together with different promoters driving cell-specific transgene expression, have been made available recently [21,30]. Here a synapsin promoter driving SynA53T gene selective expression in neurons without infecting glial cells has been chosen [21]. This choice was based on an attempt to best mimic what is currently known about PD pathophysiology, a two-step process where there is an initial loss of dopaminergic neurons as a result of α-syn aggregation, later followed by microglial-driven proinflammatory responses, which in turn enhance and self-perpetuate dopaminergic neuronal damage [20]. In other words and when compared to using rAAVs encoding SynA53T under the control of constitutive promoters [23,24,25,26,27,28], where we wanted to make sure that the initial insult with mutated α-syn specifically transduced only neurons and not glial cells.

### 3.2. Targeting the GCase-Synuclein Pathway for the Treatment of Synucleinopathy

At present, there is a broad consensus in appointing GCase as a promising target for the treatment of neurodegenerative disorders caused by the progressive aggregation of α-syn. Despite the limited knowledge on the ultimate mechanisms linking GCase loss-of-function with α-syn aggregation—and vice versa—it seems clear that any attempt focused on enhancing GCase lysosomal activity may play a beneficial effect regarding α-syn clearance, therefore, resulting in dopaminergic cell neuroprotection and ultimately leading to lower disease progression rates. Moreover, the target population would comprise all types of PD patients, with and without GBA1 mutations. It has been estimated that harboring a GBA1 mutation implies an increased risk of suffering from PD and DLB [31]. Furthermore, a natural decline in GCase activity with aging has also been reported [14]. This finding also points to the direction of increased PD risk, bearing in mind that advanced age is the main risk factor for PD and related synucleinopathies. Regarding the PD clinical phenotype, only minor differences have been observed when comparing GBA1 mutation carriers and noncarriers. Although a slightly earlier disease onset has been described for PD patients harboring GBA1 mutations, these patients face an overall more aggressive disease course [9,32,33]. Within this more aggressive disease course, a higher incidence of cognitive impairment and the presence of neuropsychiatric symptoms have been reported [6,9,34], likely reflecting earlier cortical dysfunction, and indeed, Lewy body pathology has been more often described in PD patients carrying a GBA1 mutation [2,35,36].

Another interesting feature of GBA1-related PD is that it seems that the type of GBA1 mutation variant also plays a role in the clinical course of the disease. From the two types of GBA1 mutation variants, N370S has been considered as a “mild” mutation, more closely related to PD than the “severe” L444P mutation, the one most often found in DLB patients [37,38,39]. Furthermore, the pathogenic role of mutation other than N370S and L444P, such as E326K (unrelated to Gaucher disease), needs to be ascertained, and indeed the presence of E326K mutation variant has been correlated with a faster progression to cognitive impairment [40,41]. For those patients harboring the N370S mutation variant, a residual GCase enzymatic activity of 32–38% has been estimated, whereas the more severe L444P mutation leaves a remaining GCase activity of only 13–24% [42,43,44], both mutation variants leading to an 80–95% decrease of the intrinsic catalytic activity of mutated GCase compared to the wild-type [45,46,47]. Considering sporadic forms of PD, a decline in GCase enzymatic activity ranging from 10% to 33% has been observed [3,12,13].

### 3.3. Available Choices for Enhancing GCase Enzymatic Activity

Available evidence showing GCase reduced enzymatic activity in PD patients with and without GBA1 mutations has paved the way for the design and implementation of several approaches sharing the common ground of enhancing GCase function as putative disease-modifying therapies. When considering GCase replacement therapies, although the administration of the recombinant enzyme has been a successful choice for the treatment of Gaucher disease [48,49], the main limitation is represented by the fact that GCase cannot enter the CNS at levels high enough to enhance lysosomal enzymatic activity due to limited penetration of recombinant GCase protein through the blood–brain barrier [50]. Moreover, using molecular GCase chaperones currently represents an appealing choice for improving GCase trafficking from the endoplasmic reticulum to the lysosomes, and indeed, the GCase chaperone ambroxol has been appointed as the best therapeutic candidate so far [51,52,53]. GCase chaperones other than ambroxol are currently being tested in preclinical studies [54,55,56], likely approaching clinical trials very soon. Furthermore, the hypothesis of ceramide accumulation as a pathogenic mechanism in PD patients harboring GBA1 mutations has paved the way for implementing neuroprotective strategies based on using inhibitors of glucosylceramide synthase [18,57]. Finally, the field of gene therapy also holds great promise as a feasible approach for enhancing GCase activity. In this regard, the simultaneous intraparenchymal delivery into the SNpc of two rAAVs, one coding for SynA53T, the other one carrying the GBA1 gene, prevented the degeneration of dopaminergic neurons in rats [15], and indeed, the recent availability of BBB-penetrant rAAV variants coding for GBA1 (rAAV9-PHP.B-GBA1) has demonstrated good performance for inducing α-syn clearance in transgenic mice when injected intravenously [16]. Besides PD and related synucleinopathies, it is also worth noting that the intraparenchymal delivery of rAAV-GBA1 in a transgenic mice model of Gaucher disease showed a reduction in misfolded protein aggregates of α-syn, tau and ubiquitin at the level of the hippocampal formation [17,18].

Regardless of the chosen approach for GCase enhancement, there are many demands needed to be fulfilled by any successful therapeutic strategy targeting the GCase-α-syn pathways, such as (i) ability to induce α-syn clearance and (ii) neuroprotective effect for dopaminergic neurons. Although data presented here succeeded in fulfilling all these requirements, before considering potential translation to clinical trials, there are two additional items required to be satisfied, namely (i) demonstration of a positive effect of early interventions with rAAV9-GBA1 after a long-term readout and (ii) efficacy of rAAV9-GBA1 in late interventions, i.e., once the neurodegenerative processes are far more advanced.

## 4. Materials and Methods

A total of 11 male C57BL/6 mice (2 months of age) and 4 young adult male Macaca fascicularis primates (bodyweight 3.4–4.1 kg) were used in this study. Tissue samples from an additional group of 5 naïve mice and 3 naïve nonhuman primates were used for control purposes when measuring (i) density of TH+ neurons in the SNpc and (ii) baseline expression levels of GCase protein. Animal handling was conducted at all times following the European Council Directive 2010/63/UE (22 September 2010) and keeping with the Spanish legislation (RD53/2013). The experimental design was approved by the Ethical Committee for Animal Testing of the University of Navarra (ref: 016-17) and the Department of Health of the Government of Navarra.

### 4.1. Viral Vector Production

The recombinant ssAAV9.hsynapsin.ASYN.A53T or ssAAV9.nGUSB.GBA1 vectors were co-transfected into a HEK293T cell line by using polyethykenimine 25 kDa (Polysciences, Warrington, PA, USA) with plasmids containing the ITR-flanked transgene construct and a plasmid containing the AAV2 rep, the AAV9 cap and the adenoviral helper gene. The supernatant was collected and treated with polyethylene glycol solution (PEG8000, 8% *v*/*v* final concentration) for 72 h post-transfection and stored for 72 h at 4 °C. The supernatant was then centrifuged at 3000 rpm for 15 min. Both supernatant and pellet containing AAV particles were collected and treated with lysis buffer (50 nM Tris/HCl, 150 nM NaCl, 2 mM MgCl_2_, 0.1% Triton X-100) and kept at −80 °C. Cells were lysed by 3 cycles of freezing/thawing. Viral particles were treated with DNAse and RNAse and purified by ultracentrifugation in an iodixanol gradient. AAV particles were then concentrated through Centricon tubes (YM-100; Millipore, Bedford, MA, USA), aliquoted and stored at −80 °C until used. Viral vector titers (viral particles vg/mL) were calculated by the qPCR method [30]. The primers used in the qPCR were (see Table 1):

### 4.2. Rodent Experiments

#### 4.2.1. Intraparenchymal Deliveries of rAAVs

Surgical anesthesia was induced by intraperitoneal injection of ketamine (100 mg/kg) and xylazine (10 mg/kg). Stereotaxic coordinates for the substantia nigra pars compacta (SNpc; 3.4 mm caudal to bregma, 1.22 mm lateral to the midline and 3.75 mm ventral to the pial surface) were calculated according to the atlas of Franklin and Paxinos [58]. Bilateral pressure-delivery of a neurospecific rAAV9-SynA53T vector was performed with a Hamilton syringe in pulses of 0.2 μL/min (1 × 10^13^ vg/mL; 1 deposit of 2 μL each into the left and right SNpc). Once injections were completed, the needle was left in place for an additional time of 10 min before withdrawal to minimize rAAV reflux through the injection tract. Four weeks later, animals were pressure-injected with the therapeutic vector rAAV9 coding for GBA1 under the control of a constitutive GusB promoter (rAAV9-GBA1; 1 × 10^13^ vg/mL; 1 deposit of 2 μL) into the right SNpc and with a control empty vector (rAAV9-null; 1 × 10^13^ vg/mL; 1 deposit of 2 μL) into the left SNpc. Coordinates and procedures for these injections were the same as previously described for rAAV9-SynA53T.

#### 4.2.2. Necropsy and Tissue Processing

Anesthesia was first induced with an intramuscular injection of ketamine (10 mg/kg) followed by a terminal overdose of chloral hydrate (100 mg/kg) and perfused transcardially with an infusion pump. The perfusates consisted of a saline ringer solution followed by 50 mL of a fixative solution made of 4% paraformaldehyde and 0.1% glutaraldehyde in 0.125 M phosphate buffer (PB) pH 7.4. Once perfusion was completed, the skull was opened, and the brains were removed and stored for 48 h in a cryoprotectant solution containing 20% glycerin and 2% dimethyl sulfoxide (DMSO) in 0.125 M PB, pH 7.4. Next, frozen coronal sections (40 mm thick) were obtained on a sliding microtome and collected in 0.125 M PB, pH 7.4, as 10 series of adjacent sections. These series were used for the immunohistochemical detection of (1) α-syn, (2) phosphorylated α-syn, (3) tyrosine hydroxylase, (4) dopamine transporter, (5) glucocerebrosidase, (6) ionized calcium-binding adaptor molecule 1 (Iba-1), (7) major histocompatibility complex class II (MHC-II) and (8–10) combined multiple immunofluorescent detections of several markers.

Single-immunoperoxidase stains for the markers listed above were carried out by overnight incubation with specific primary antisera, followed by incubation with the corresponding biotinylated IgGs for 120 min, followed by a Vectastain avidin–biotin complex for 60 min. Final visualization was carried out with a diaminobenzidine-H_2_O_2_ reaction for 5–10 min. Sections were mounted on gelatin-coated slides, air-dried, dehydrated and cleared in toluene and finally coverslipped with Entellan (Merck, Darmstadt, Germany). A complete list of antibodies used, indicating antibody dilutions, incubation times and commercial sources, is provided in Table 2.

### 4.3. Nonhuman Primate Experiments

#### 4.3.1. Intraparenchymal Deliveries of rAAVs

Surgical anesthesia was induced by intramuscular injection of ketamine (5 mg/kg) and midazolam (5 mg/kg). Local anesthesia was implemented just before surgery with a 10% solution of lidocaine. Analgesia was achieved with a single intramuscular injection of flunixin meglumine (Finadyne, 5 mg/kg) delivered at the end of the surgical procedure and repeated 24 and 48 h post-surgery. A similar schedule was conducted for antibiotic delivery (ampicillin, 0.5 mL/day). After surgery, nonhuman primates (NHPs) were kept under constant monitoring in single cages with ad libitum access to food and water.

Stereotaxic coordinates for the SNpc were taken from the atlas of Lanciego and Vázquez [59]. During surgery, target selection was assisted by ventriculography. Bilateral pressure-deliveries of a neurospecific rAAV9-SynA53T vector was made through a Hamilton syringe in pulses of 1 μL/min for a total volume of 5 μL each (1 × 10^13^ vg/mL) into two sites in the SNpc, each deposit spaced 1 mm in the rostrocaudal direction to transduce the highest possible extent of the SNpc. Once injections were completed, the needle was left in place for an additional time of 10 min before withdrawal to minimize reflux through the injection tract. Coordinates for the more rostral SNpc injection of rAAV9-SynA53T were 7.5 mm caudal to the anterior commissure (ac), 5 mm ventral to the bicommissural plane (ac–pc plane) and 4 mm lateral to the midline, whereby the more caudal deposit was placed 8.5 mm caudal to ac, 5.5 mm ventral to the ac–pc plane and 4 mm lateral to the midline. Four weeks later, animals were pressure-injected with the therapeutic vector rAAV9-GBA1 (GusB promoter; 1 × 10^13^ vg/mL; two sites, 10 μL each) into the left SNpc and with a control empty vector (rAAV9-null; 1 × 10^13^ vg/mL; two sites, 10 μL each) into the right SNpc. Coordinates and procedures for these injections were the same as previously described for rAAV9-SynA53T deliveries.

#### 4.3.2. MicroPET Scans

MicroPET scans with ^11^C-dihydrotetrabenazine (^11^C-DTBZ), a selective VMAT2 ligand) were performed on each animal at baseline and 4, 8 and 12 weeks post-injection of rAAV9-SynA53T. ^11^C-DTBZ was synthesized at the Department of Nuclear Medicine, Clinica Universidad de Navarra, with a radiochemical purity of >95% following available protocol [60]. MicroPET images were acquired on a dedicated small animal Philips mosaic tomograph (Cleveland, OH, USA). The standard acquisition and quantification of MicroPET studies with ^11^C-DTBZ were conducted as previously described [61]. In brief, a dynamic MicroPET study of 40 min was acquired after the intravenous injection of the radiotracer. MicroPET studies were analyzed by a suitable tracer kinetic model using PMOD v3.2 software (PMOD Technologies Ltd., Adliswil, Switzerland) to obtain parametric images containing the information on the binding potential of the VMAT2 transporter. Parametric images were spatially normalized into standard stereotaxic space using a specific template [62]. The binding potential was measured using a predefined map of regions of interest (ROIs) defined over MRI images comprising the caudate and putamen nuclei. Changes in radiotracer binding potential were calculated for each animal at each time point.

#### 4.3.3. Necropsy and Tissue Processing

Anesthesia was first induced with an intramuscular injection of ketamine (10 mg/kg), followed by a terminal overdose of chloral hydrate (100 mg/kg) and perfused transcardially with an infusion pump. The perfusates consisted of a saline Ringer solution followed by 3000 mL of a fixative solution made of 4% paraformaldehyde and 0.1% glutaraldehyde in 0.125 M PB pH 7.4. Perfusion was continued with 1000 mL of a cryoprotectant solution containing 10% glycerin and 1% DMSO in 0.125 M PB pH 7.4. Once perfusion was completed, the skull was opened and the brain removed and stored for 48 h in a cryoprotectant solution containing 20% glycerin and 2% DMSO in 0.125 M PB pH 7.4. Next, frozen coronal sections (40 μm-thick) were obtained on a sliding microtome and collected in 0.125 M PB pH 7.4 as 10 series of adjacent sections. Conducted immunohistochemical processing of NHP brain tissue samples was the same as described above for experiments carried out in mice samples.

#### 4.3.4. Unbiased Stereological Estimation of TH+ Neurons in the SNpc

Stereological estimation of TH+ neurons was performed in all animals using an optical fractionator unbiased sampling design. Up to seven equally spaced coronal sections taken through the entire rostrocaudal extent of the SNpc were sampled per animal. Stereological analysis was performed using an Olympus Bx61 microscope (Olympus, Hicksville, NY) equipped with a DP71 digital camera (Olympus) and a motorized X-Y-Z stepper and with the NewCast Visiopharm software version 3.6.0.0 (Horsholm, Denmark). The analyzed regions from the left and right SNpc territories were outlined to allow magnification. A counting frame was superimposed on the image, and the neurons were sampled at high magnification (Plan Apo oil-immersion 100× lens, N.A. 1.4) with the nucleolus used as sampling unit (Nissl-counterstained sections processed for TH immunohistochemistry) and up to 200 cells were counted in each specimen. The percentage of neuronal loss in the SNpc was calculated against the values obtained in naïve animals (five mice and three NHPs). One-way analysis of variance (ANOVA) with repeated measures followed by Tukey’s post hoc test was used to estimate the overall significance. Probability (*p*) values less than 0.05 were considered to be statistically significant. SPSS 15.0 software was used.

#### 4.3.5. Unbiased Estimation of α-Syn+ Neurons in the SNpc

The number of α-syn+ and phospho-α-syn+ neurons in the SNpc was performed in all animals using a deep-learning, artificial intelligence algorithm known as Aiforia (www.aiforia.com). Up to nine to ten equally spaced coronal sections covering the whole rostrocaudal extent of the SNpc were sampled per animal. Two different dedicated algorithms (one for α-syn and another one for phospho-α-syn) were prepared, trained and validated, resulting in an error of 1.2% for α-syn+ cells counting and of 1.8% for quantifying phospho-α-syn+ neurons. Left and right SNpc territories were outlined at low magnification, excluding neighboring areas, such as the ventral tegmental area and the retrorubral field. Sections processed for α-syn and phosphor-α-syn were scanned at 20× in an Aperio CS2 scan (Leica, Wetzlar, Germany) and uploaded to Aiforia cloud. Once validated, the dedicated algorithms were released and used as templates for quantifying labeled cells in the left and right SNpc. Obtained data are presented as mean plus the standard deviation (SD), and statistical significance is calculated using an unpaired *t*-test analysis by software GraphPad Prism version 9.0.2 for Windows. The threshold for statistical significance was set as *p* < 0.05.

#### 4.3.6. Estimation of GCase Expression Levels by Optical Density

Optical density (OD) of GCase-stained in the SNpc was measured in all four animals treated with rAAV9-GBA1 and rAAV9-null vectors and compared to baseline GCase expression levels gathered from three naïve animals here used for control purposes. In brief, up to 9 to 10 equally spaced coronal sections taken through the full rostrocaudal extent of the SNpc were scanned at 20× in an Aperio CS2 (Leica). Left and right SNpc territories were outlined at low magnification, excluding neighboring areas, such as the ventral tegmental area and the retrorubral field. GCase OD was measured in these sections with Fiji ImageJ software. Obtained data are presented as mean plus the standard deviation (SD), and statistical significance is calculated using an unpaired *t*-test analysis by software GraphPad Prism version 9.0.2 for Windows. The threshold for statistical significance was set as *p* < 0.05.

## 5. Patents

Some data results from work presented here were included in the patent entitled “Viral particles for use in treating synucleinopathies, such as Parkinson’s diseases by gene therapy” (reference WO 2021/028299 A1).

## Figures and Tables

**Table 1:** qPCR primers.

Forward-ITR	5′-GGAACCCCTAGTGATGGAGTT-3′
Reverse-ITR	5′-CGGCCTCAGTGAGCGA-3′
Forward-ASYN	5′-AGAAGACAGTGGAGGGAGCA-3′
Reverse-ASYN	5′TGTCAGGATCCACAGGCATA-3′
Forward-GBA	5′-CCTGGATGCTTATGCTGAGC-3′
Reverse-GBA	5′-CCAGATACCAGTGCACAGCAATC-3′

**Table 2:** List of primary and secondary antibodies.

Antibody	Dilution	Incubation Time	Supplier	Reference
Goat anti-tyrosine hydroxylase	1:500	overnight	MyBioSource	MBS421729
Rabbit anti-tyrosine hydroxylase	1:100	overnight	Millipore	AB152
Mouse anti-glucocerebrosidase	1:500	overnight	Abcam	Ab55080
Rabbit anti-glucocerebrosidase	1:500	overnight	Thermo Fisher	PAS-21347
Goat anti-α-synuclein	1:300	overnight	Abcam	Ab2080
Mouse anti-α-synuclein	1:500	overnight	Abcam	Ab27766
Mouse anti-phospho-α-synuclein	1:1000	overnight	Wako	015-25291
Rat anti-dopamine transporter	1:500	overnight	Millipore	MAB369
Donkey anti-goat IgG biotinylated	1:600	120 min	Jackson	705-065-147
Donkey anti-rabbit IgG biotinylated	1:600	120 min	Jackson	711-065-152
Donkey anti-mouse IgG biotinylated	1:600	120 min	Jackson	715-066-150
Goat anti-rat IgG biotinylated	1:600	120 min	Jackson	112-066-003
Vectastain ABC HRP standard kit	1:1000	60 min	Vector Labs	PK-4000

## Data Availability

Data reported here are available from authors upon reasonable request.
